# Filaggrin-null mutations are associated with increased maturation markers on Langerhans cells

**DOI:** 10.1016/j.jaci.2015.11.040

**Published:** 2016-08

**Authors:** Claire S. Leitch, Eenass Natafji, Cunjing Yu, Sharizan Abdul-Ghaffar, Nayani Madarasingha, Zoë C. Venables, Roland Chu, Paul M. Fitch, Andrew J. Muinonen-Martin, Linda E. Campbell, W.H. Irwin McLean, Jürgen Schwarze, Sarah E.M. Howie, Richard B. Weller

**Affiliations:** a Department of Dermatology, Royal Infirmary of Edinburgh, Edinburgh, United Kingdom; b MRC Centre for Inflammation Research, University of Edinburgh, Edinburgh, United Kingdom; c School of Chemistry, University of Edinburgh, Edinburgh, United Kingdom; d Alan Lyell Centre for Dermatology, Southern General Hospital, Glasgow, United Kingdom; e Centre for Dermatology and Genetic Medicine, University of Dundee, Dundee, United Kingdom

**Keywords:** Filaggrin, atopic dermatitis, Langerhans cells, urocanic acid, costimulatory molecules, AD, Atopic dermatitis, APC, Antigen-presenting cell, FACS, Fluorescence-activated cell sorting, FITC, Fluorescein isothiocyanate, *FLG*, Filaggrin gene, FoxP3, Forkhead box protein 3, IV, Ichthyosis vulgaris, LC, Langerhans cell, LTA, Lipotechoic acid, MDCC, Monocyte-derived dendritic cell, MFI, Mean fluorescence intensity, PD-L1, Programmed death ligand 1, PE, Phycoerythrin, PerCP, Peridinin-chlorophyll-protein complex, SASSAD, Six Area, Six Sign Atopic Dermatitis Score, TEWL, Transepidermal water loss, UCA, Urocanic acid, WT, Wild-type

## Abstract

**Background:**

Mutations in the gene encoding filaggrin *(FLG)*, an epidermal structural protein, are the strongest risk factor identified for the development of atopic dermatitis (AD). Up to 50% of patients with moderate-to-severe AD in European populations have *FLG*-null alleles compared with a general population frequency of 7% to 10%.

**Objective:**

This study aimed to investigate the relationship between *FLG*-null mutations and epidermal antigen-presenting cell (APC) maturation in subjects with and without AD. Additionally, we investigated whether the *cis* isomer of urocanic acid (UCA), a filaggrin breakdown product, exerts immunomodulatory effects on dendritic cells.

**Methods:**

Epidermal APCs from nonlesional skin were assessed by using flow cytometry (n = 27) and confocal microscopy (n = 16). Monocyte-derived dendritic cells from healthy volunteers were used to assess the effects of *cis-* and *trans-*UCA on dendritic cell phenotype by using flow cytometry (n = 11).

**Results:**

Epidermal APCs from *FLG*-null subjects had increased CD11c expression. Confocal microscopy confirmed this and additionally revealed an increased number of epidermal CD83^+^ Langerhans cells in *FLG*-null subjects. *In vitro* differentiation in the presence of *cis*-UCA significantly reduced costimulatory molecule expression on monocyte-derived dendritic cells from healthy volunteers and increased their ability to induce a regulatory T-cell phenotype in mixed lymphocyte reactions.

**Conclusions:**

We show that subjects with *FLG*-null mutations have more mature Langerhans cells in nonlesional skin irrespective of whether they have AD. We also demonstrate that *cis*-UCA reduces maturation of dendritic cells and increases their capacity to induce regulatory T cells, suggesting a novel link between filaggrin deficiency and immune dysregulation.

Mutations in the gene encoding filaggrin *(FLG)*, an epidermal structural protein, are associated with different phenotypes: atopic dermatitis (AD), ichthyosis vulgaris (IV), or clinically normal skin. Up to 50% of patients with moderate-to-severe AD in European populations have at least 1 *FLG*-null allele^1^ compared with a general population frequency of 7% to 10%.^2^ Although the finding of such a robust gene association has enlivened research into what had been considered a complex polygenic disorder, the relationship between *FLG*-null mutations and AD has not yet been clearly elucidated.

The “outside-inside” theory of the pathogenesis of AD proposes that a deficient skin barrier is the primary abnormality driving the disease, allowing allergens, antigens, and microbial danger signals to penetrate the epidermis and activate local antigen-presenting cells (APCs). Human epidermis is colonized by a specialized subset of APCs known as Langerhans cells (LCs). In patients with AD, persistent LC stimulation through a defective skin barrier can result in chronic T_H_2-driven atopic inflammation.^3^ Because filaggrin is involved in collapsing keratinocytes to form the densely packed stratum corneum, it is thought to play a crucial role in maintaining physical skin barrier integrity.^4^, ^5^ Therefore the outside-inside theory suggests a causative link between genetic filaggrin deficiency and the development of AD.

Filaggrin breakdown products might also be important in skin barrier function. Filaggrin is rich in histidine, which is converted in the epidermis to *trans-*urocanic acid (*trans*-UCA). This in turn is naturally converted to *cis*-UCA in the skin on exposure to UV radiation.^6^ *cis*-UCA has been previously investigated as a potential immunomodulator of allergic responses in the skin, and it has been shown that topical^7^ or systemic^8^ administration of *cis*-UCA suppresses skin immune responses to viral infection in mice. Given systemically to mice, it induces tolerance to cutaneous allergens,^9^ whereas in human subjects topical application of *cis*-UCA blunts the cell-mediated response to dinitrochlorobenzene.^10^ Although topical *cis*-UCA is currently in early-phase trials as a possible treatment for AD,^11^ its exact mode of action is not known.

In this study we investigated the maturation state of epidermal APCs in subjects with *FLG*-null mutations, looking for any differences in APC phenotype between *FLG*-null subjects with and without AD and any correlation with skin barrier function. We also investigated whether *cis*-UCA affects dendritic cell phenotype and function *in vitro*.

## Methods

### Recruitment and characterization of volunteers

The study was approved by the Lothian Research Ethics Committee (07/MRE00/109), and informed written consent was obtained from all participants. Subjects were recruited from general outpatient and patch test clinics at the Department of Dermatology, Royal Infirmary of Edinburgh (patients with AD only), and medical students and staff at the University of Edinburgh and the Southern General Hospital Glasgow. Diagnosis of AD was based on the UK Working Party's Diagnostic Criteria.^12^ Subjects were interviewed about personal and family history of atopy and current and past management of AD and were assessed for symptoms and signs of AD and IV by means of both questionnaire and examination.^13^ AD severity was measured by using the Six Area, Six Sign Atopic Dermatitis severity score.^14^ No subjects had used oral or topical steroids for at least 1 week before the study. Whole blood was obtained from heathy volunteers at the MRC Centre for Inflammation Research, University of Edinburgh (ethics approval 08/S1103/38).

### Genotyping

Genotyping of subjects involved in physical skin barrier assessment and suction blister analysis by flow cytometry was performed with a TaqMan-based allelic discrimination assay (Applied Biosystems, Foster City, Calif) for the 2 most common *FLG* mutations in European populations (2282del4 and R501X). Samples were analyzed at Source Bioscience (Nottingham, United Kingdom) or by the Human Genetics Unit, University of Dundee. Subjects involved in suction blister analysis using confocal microscopy were genotyped for the 4 most common *FLG* mutations in European populations (2282del4, R501X, S3247X, and R2447X) at the Wellcome Trust Clinical Research Facility, Western General Hospital, Edinburgh, Scotland. Probes and primers were as described previously.^15^

### Suction blisters

Suction blister cups were applied to the upper inner arm to produce epidermal blisters with a suction blister device (InnoKas Medical Oy, Kempele, Finland). Each cup formed up to 5 blisters 5 mm in diameter after 90 to 120 minutes at a suction pressure of 400 mbar applied at 10-second intervals. Suction blisters from patients with AD were taken from clinically uninvolved skin. For details of the quantification of *cis*- and *trans*-UCA isomers in suction blister fluid, see the Methods section in this article's Online Repository at www.jacionline.org.

### Measurement of transepidermal water loss

Subjects were asked not to take antihistamines or apply any topical treatments, including emollients, for 3 days before the study. All physiologic measurements were taken from uninvolved flexor forearm skin (4 cm below the antecubital fossa) after 10 minutes of acclimatization to standardized conditions (20°C-22°C; humidity, 40% to 60%). Measurements were performed in an open-top box to limit air convection currents and condensation.^16^ Transepidermal water loss (TEWL) was measured with the Tewameter TM300 (Courage and Khazaka, Cologne, Germany) with an open-chamber probe. Measurements were repeated 3 times.

### Tape stripping

Tape stripping was performed with 14-mm D-Squame tape discs (CuDerm, Dallas, Tex). A cylindrical weight applying a pressure of 225 g/cm^2^ was used for 10 seconds before the tapes were removed unidirectionally with forceps.^17^ The number of tape strips required to abrogate the permeability barrier (TEWL >20 g/m^2^/h)^18^ was recorded by measuring TEWL after each tape strip.

### Phenotypic analysis of epidermal APCs

For flow cytometry, blister roofs were incubated at 37°C for 30 minutes with 0.05% trypsin in PBS before manual disaggregation. PBS containing 1% FCS was added, and the cells were washed by means of centrifugation (10 minutes at 300*g*). The pellet was resuspended in 1 mL of fluorescence-activated cell sorting (FACS) wash buffer (BD Biosciences, Oxford, United Kingdom) containing 5% mouse serum and incubated for 10 minutes on ice, washed, and resuspended in 200 μL of FACS wash containing 5% mouse serum. For details of antibody staining, please see the Methods section in this article's Online Repository. Samples were collected with a BD LSR Fortessa Flow cytometer (BD Biosciences). Anti-mouse CompBead Plus (BD Biosciences) was used to calibrate color compensation. Results were analyzed with FlowJo software (TreeStar, Ashland, Ore).

For confocal microscopy, epidermal samples were fixed for 30 minutes in 90% acetone/10% methanol and then washed in 3 changes of PBS (Gibco, Grand Island, NY) for 10 minutes each. Samples were incubated with antibodies for 1 hour in PBS with 0.1% BSA (Sigma-Aldrich, St Louis, Mo) and then washed 3 times in PBS. For details of antibody staining panels, see the Methods section in this article's Online Repository. Staining was done at room temperature, and samples were protected from light. Samples were mounted on Superfrost Plus slides (BDH Laboratory Supplies, Dorset, United Kingdom) in Permafluor (Thermo Scientific, Waltham, Mass) and stored at 2°C to 5°C.

Slides were observed with a Leica SP5 confocal microscope (Leica Microsystems, Wetzlar, Germany) with a ×40 oil immersion objective. Image stacks were acquired in 1.01-μm slices through the epidermis. Images were deconvolved with Huygens Essential Software (Scientific Volume Imaging, Hilversum, The Netherlands). Basic image analysis was performed with ImageJ software (National Institutes of Health, Bethesda, Md), and 3-dimensional image analysis was performed with Volocity 5.5 (PerkinElmer, Waltham, Mass). LC volumes were calculated by using CD1a staining as a proxy measure. Individual cells were identified by using an intensity threshold, and 50 cell volumes were averaged to produce a mean LC volume for each subject. The cell volume enclosed by CD11c cell-surface expression was also calculated as above.

### Generation of monocyte-derived dendritic cells

PBMCs were obtained from whole blood by means of Ficoll gradient centrifugation (Ficoll-Paque PLUS; GE Healthcare, Buckinghamshire, United Kingdom). CD14^+^ monocytes were isolated by means of positive selection by using magnetic-activated cell sorting with CD14^+^ microbeads, according to the manufacturer's instructions (Miltenyi Biotec, Bergisch Gladbach, Germany). CD14^+^ monocytes were cultured in 12-well plates (Nunclon Delta surface, Thermo Scientific) at a density of 2 × 10^6^ cells/mL in RPMI 1640 culture medium (Gibco) buffered with 20 mmol/L HEPES, 5% human AB serum, and 1% l-glutamine for 7 days. Medium was supplemented with 50 ng/mL GM-CSF (PeproTech, Rocky Hill, NJ) and 15 ng/mL IL-4 (Invitrogen, Carlsbad, Calif), and *cis*- or *trans*-UCA (Sigma-Aldrich) was added to wells at concentrations of 10 or 100 μg/mL on day 0. These concentrations were chosen for the *in vitro* experiments to best mimic physiologic concentrations.^19^ Fresh medium supplemented with cytokines and UCA was added on days 3 and 5. A portion of the immature dendritic cells was collected on day 7 for analysis; remaining cells were stimulated with LPS (1 ng/mL) or lipotechoic acid (LTA) (10 μg/mL) and harvested on day 8 when monocyte-derived dendritic cell (MDCC) viability was at least 90% in cultures with and without *cis*- and *trans*-UCA. All cultures were protected from light.

### Flow cytometric analysis of MDDCs

For details of antibody staining panels, see the Methods section in this article's Online Repository.

### MDCC and CD4 T-cell coculture

Immature MDCCs conditioned with or without 100 μg/mL *cis*-UCA were cocultured with allogeneic CD4 T cells stained with proliferation dye EF670 (eBioscience, San Diego, Calif) at a ratio of 1:10 in 96-well plates. CD4 T-cell isolation details are presented in the Methods section in this article's Online Repository. Cells were cocultured at 37°C in a 5% CO_2_ humidified atmosphere for 5 days. Anti-CD3/CD28 (1 μg/mL, eBioscience) was added to a portion of CD4 T cells as a positive control. The proliferation of CD4 T cells and the proportion of CD4^+^CD25^+^ forkhead box protein 3 (FoxP3)^+^ CD127^−^ cells were analyzed by using flow cytometry. For details of antibody staining, see the Methods section in this article's Online Repository.

### Statistical analysis

Statistical analysis was performed with Prism 6 software (GraphPad Software, San Diego, Calif). Mann-Whitney *U*, Kruskal-Wallis, and Friedman tests with Dunn posttest comparisons were used to analyze data. Data are presented as means ± SDs, unless otherwise stated. *P* values of less than .05 were considered significant.

## Results

### Subjects

Two hundred sixty-four subjects were genotyped. Of these, 117 were clinically phenotyped and participated in further studies. Seventy-seven had AD (all with mild-to-moderate disease), of whom 56 were wild-type (WT) and 21 had *FLG*-null mutations (18 *FLG* heterozygote [*FLG*^+/-^] and 3 *FLG* homozygote [*FLG*^−/−^]). The remaining 40 subjects had clinically normal skin; 27 were WT, and 13 had *FLG*-null mutations (all *FLG*^+/−^). No subjects met the diagnostic criteria for IV.

Mean Six Area, Six Sign Atopic Dermatitis scores were very similar between AD groups involved in each part of the study (Table I). Total serum IgE levels (in international units per milliliter) were measured in 78 of the 84 subjects who had TEWL measured and tape-stripping performed. As expected, IgE levels were significantly higher in subjects with AD (629.5 ± 1286 IU/mL) compared with those in subjects without AD (54 ± 59.3 IU/mL, *P* < .0001). However, *FLG* status did not influence IgE levels in AD subjects as there was no significant difference in IgE levels between WT subjects with AD (678 ± 1374 IU/mL) and *FLG*-null subjects with AD (443 ± 892 IU/mL, *P* = .62).

### LCs have higher CD11c expression in *FLG*-null subjects with and without AD

**Fig E1:**
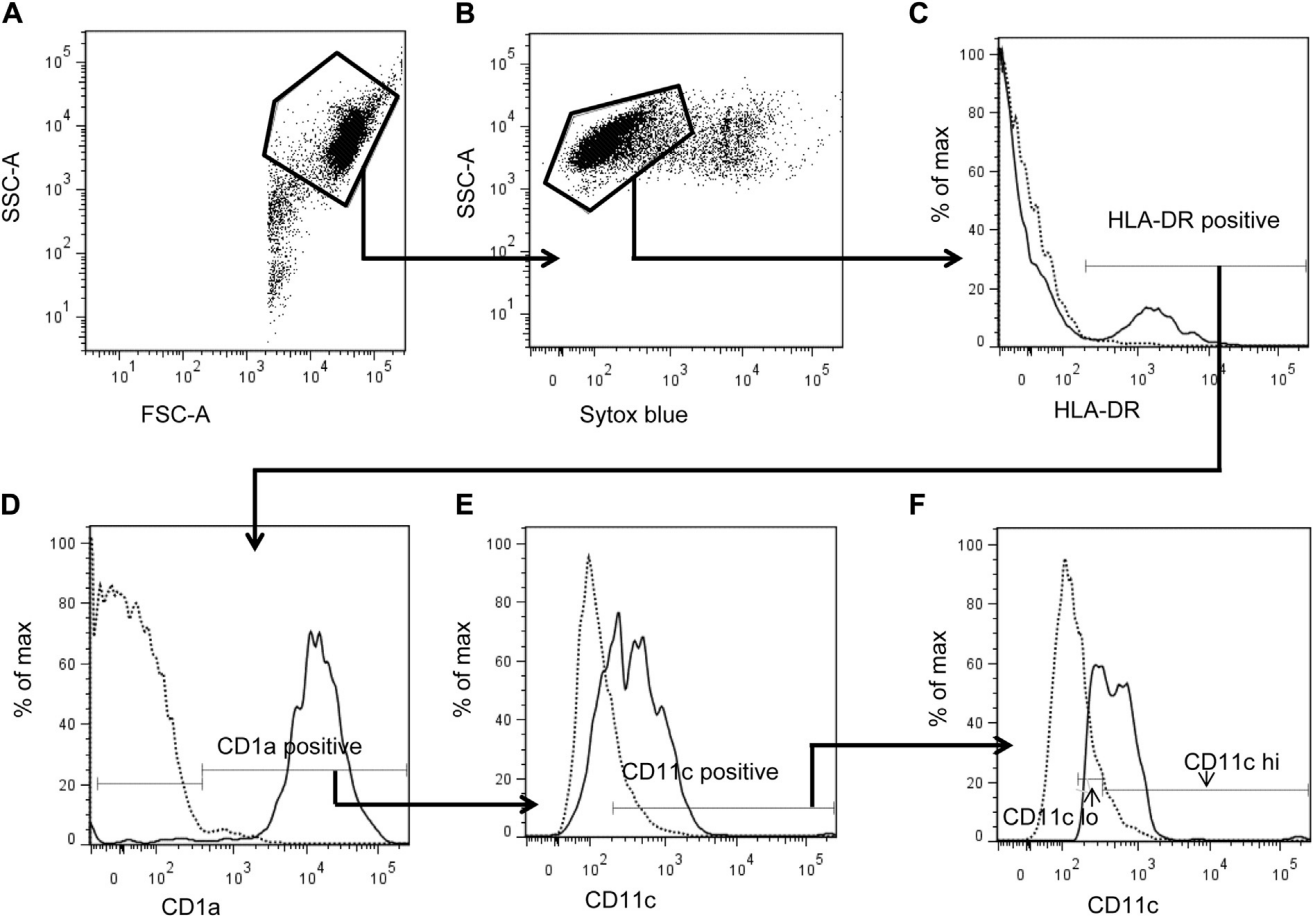
Gating strategy for single-cell suspensions created from epidermal suction blister roofs. Representative FlowJo cytometry plots are shown. **A,** Forward scatter *(FSC)*–A, side scatter *(SSC)*–A plot gated to exclude subcellular debris. **B,** SYTOX Blue SSC-A plot gated on live cells. **C,** HLA-DR: percentage of the maximum histogram showing all epidermal cells gated on HLA-DR positivity. **D,** CD1a: percentage of the maximum histogram showing HLA-DR^+^ cells gated on CD1a positivity. **E,** CD11c: percentage of the maximum histogram showing HLA-DR^+^ and CD1a^+^ cells gated on CD11c positivity. **F,** CD11c expression was divided into low and high groups to reflect the normal low expression of CD11c by LCs.

**Fig 1:**
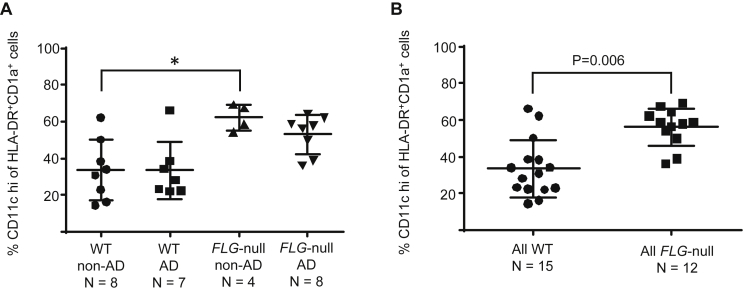
*FLG*-null subjects with and without AD have a higher proportion of CD11c^hi^ epidermal APCs than WT subjects. **A,** Scatter plot comparing the percentage of CD11c^hi^ cells among the different subject groups. *P* = .009, Kruskal-Wallis test. *P < .05, Dunn multiple comparison posttest *P* values. **B,** Scatter plot comparing the percentage of CD11c^hi^ cells in all WT subjects versus all *FLG*-null subjects. *P* values were calculated by using the Mann-Whitney test, as shown on the graph.

Twenty-seven subjects had suction blister samples analyzed by flow cytometry (for gating strategy, see Fig E1 in this article's Online Repository at www.jacionline.org). Total live cell yields ranged from 5,000 to 65,000, without significant differences between groups. There was no difference in the time taken to raise blisters within the different subject groups. Proportions of HLA-DR^+^CD1a^+^ cells were similar between the groups (2.9% to 4.3%). There was a significantly higher proportion of CD11c^hi^ cells among HLA-DR^+^CD1a^+^ cells in *FLG*-null than in WT subjects, with the highest level in *FLG*-null subjects without AD (all *FLG*-null subjects: 56% ± 10.2% CD11c^hi^ cells vs all WT subjects: 34% ± 15.6%, *P* = .0006, Fig 1).

**Fig E2:**
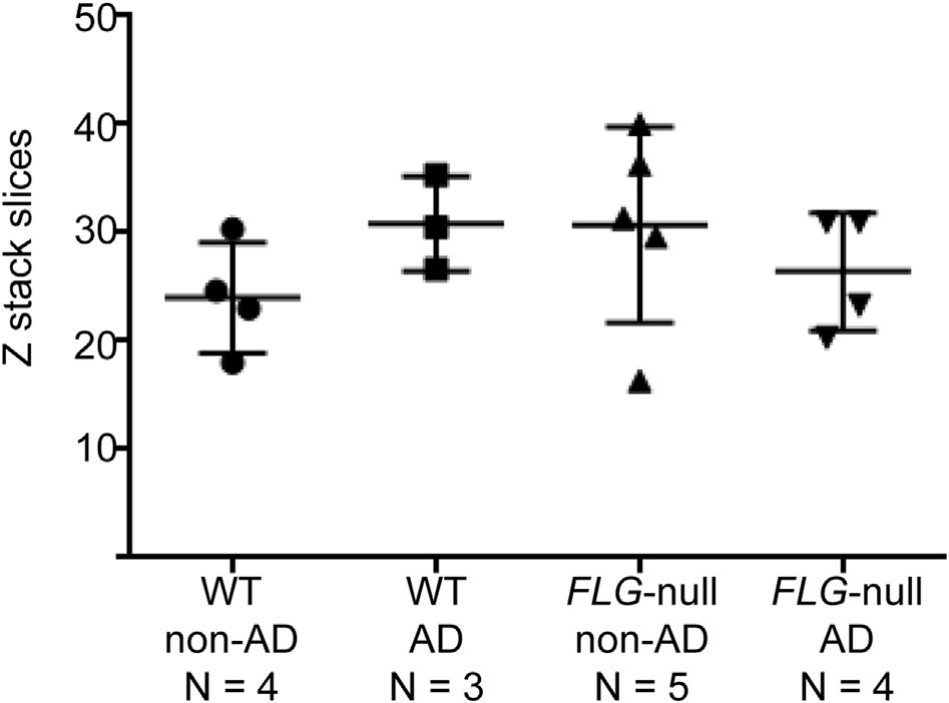
Epidermal thickness of suction blister roof samples. The number of 1.01-μm z-stack slices needed to visualize each blister roof specimen was calculated as a proxy measure of epidermal thickness. This scatter dot plot shows mean values for each volunteer averaged from counts from 3 different stacks. *Lines* show means and SDs. *P* = .34, Kruskal-Wallis test.

**Fig 2:**
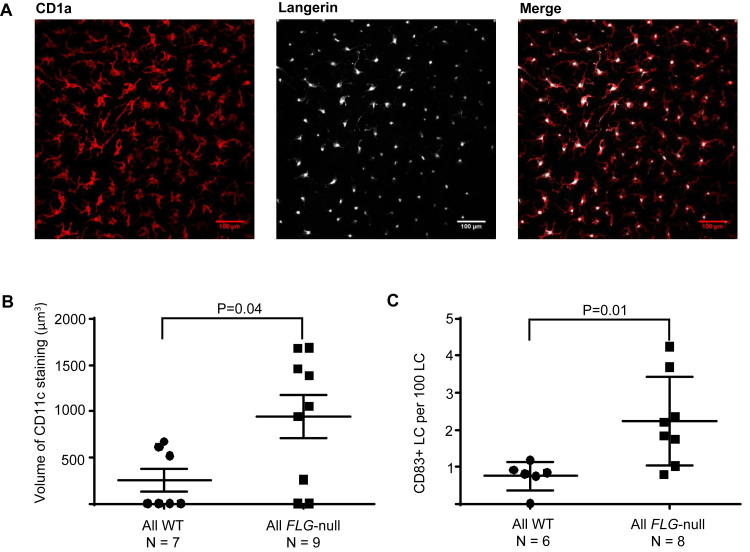
Confocal microscopy confirms increased CD11c expression in LCs in *FLG*-null subjects with and without AD. **A,** CD1a^+^*(red)* and langerin *(white)* measurement. **B,** Scatter plot comparing the mean volume of CD11c staining per LC in all WT subjects versus all *FLG*-null subjects. *P* values were calculated by using the Mann-Whitney test. **C,** Scatter plot comparing the mean number of CD83^+^ LCs per 100 LCs in all WT subjects versus all *FLG*-null subjects. *P* values were calculated by using the Mann-Whitney test.

Sixteen subjects had suction blister samples analyzed by using confocal microscopy. There was no significant difference in epidermal thickness between the different groups, as calculated by the mean number of z-stack slices required to visualize each sample (see Fig E2 in this article's Online Repository at www.jacionline.org). All CD1a^+^ dendritic cells in the blister roofs were langerin positive, confirming that they were all LCs (Fig 2, *A*). The mean number of LCs per square millimeter, the mean volume of LCs, and the mean number of dendrites per LC were similar between the groups (data not shown). Comparing all *FLG*-null subjects with all WT subjects, the mean volume enclosed by CD11c staining (in cubic micrometers) was significantly higher in *FLG*-null subjects (all *FLG*-null subjects: 941.2 ± 690.9 μm^3^ vs all WT subjects: 257.5 ± 324.3 μm^3^, *P* = .04; Fig 2, *B*).

### More CD83^+^ LCs in *FLG*-null subjects with and without AD

**Fig E3:**
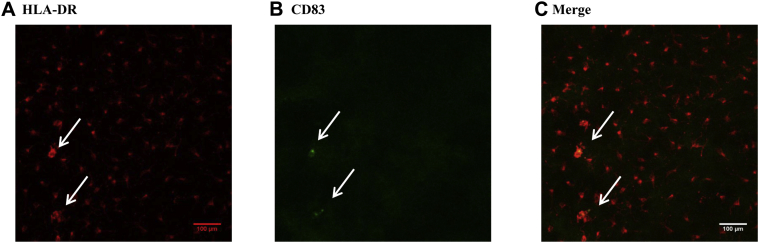
CD83 and HLA-DR staining of epidermal suction blister roofs. Intact suction blister roofs from nonlesional skin were incubated with antibodies to CD1a, CD11c, CD83, HLA-DR, and langerin/CD207. **A,** HLA-DR staining of epidermal dendritic cells *(red)* in a representative sample. Note that occasional cells express more HLA-DR *(arrows)*. **B,** CD83 staining showing occasional CD83^+^ cells in the same sample (*green*, *arrows*). **C,** Merged image showing that cells expressing more HLA-DR are also CD83^+^*(arrows)*.

Analysis of confocal microscopy images revealed occasional CD83^+^ LCs, which also showed increased expression of HLA-DR (see Fig E3 in this article's Online Repository at www.jacionline.org). Compared with all WT subjects, the number of CD83^+^ LCs was significantly higher in *FLG*-null subjects (all *FLG*-null subjects: 2.23 ± 1.2 CD83^+^ LCs per 100 LCs vs all WT subjects: 0.75 ± 0.4 CD83^+^ LCs per 100 LCs, *P* = .01; Fig 2, *C*). There were no significant differences in the mean volume enclosed by HLA-DR staining between groups (data not shown).

### *FLG* status only affects barrier function in patients with AD

**Fig 3:**
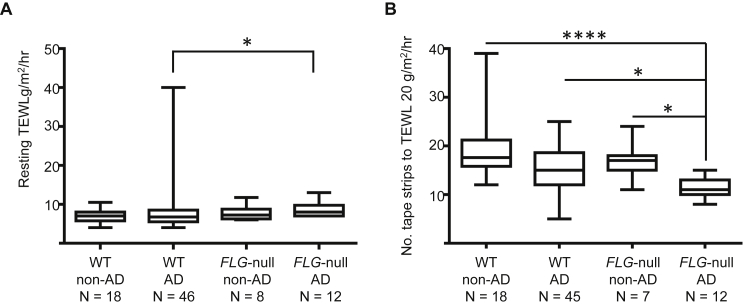
Skin barrier function is reduced in *FLG*-null subjects with but not without AD. **A,** Comparison of mean resting TEWL in all subject groups. *P* = .02, *FLG*-null subjects with AD versus WT subjects with AD, Mann-Whitney test. *Lines* show minimum to maximum values. **B,** Plot showing the number of tape strips required to reach a TEWL of greater than 20 g/h/m^2^ in all subject groups. *P* ≤ .0001, Dunn multiple comparison posttest values shown on graph. *P < .05 and ****P < .0001. *Lines* show minimum to maximum values.

Resting TEWL was measured in 84 subjects, and the number of tape strips required to reach a TEWL of greater than 20 g/m^2^/h was measured in 82 subjects. TEWL was significantly higher in the *FLG*-null AD group than in the WT AD group (*FLG*-null AD: 8.7 ± 1.9 g/m^2^/h vs WT AD: 8.1 ± 5.6 g/m^2^/h, *P* = .02; Fig 3, *A*). Two WT subjects with AD with very high resting TEWL of greater than 2 SDs from the mean were identified as statistical outliers by using a Robust regression and Outlier remover (ROUT) test (Q = 0.2%). When these subjects were excluded from the analysis, the mean TEWL in the WT AD group was 7.1 ± 2.3 g/m^2^/h (*P* = .007, *FLG*-null AD vs WT AD groups). The number of tape strips required to reach a TEWL of greater than 20 g/m^2^/h per hour was significantly lower in the *FLG*-null AD group compared with all other groups (*FLG*-null AD: 11.25 ± 2 strips vs WT AD: 14.7 ± 4.9 strips vs *FLG*-null non-AD: 17.00 ± 3.9 strips vs WT non-AD: 18.9 ± 5.9 strips, *P* = .0001; Fig 3, *B*). Interestingly, subjects without AD with and without *FLG*-null mutations required a similar number of tape strips to reduce the permeability barrier.

### *Cis*-UCA reduces costimulatory molecule expression in MDCCs

**Fig E4:**
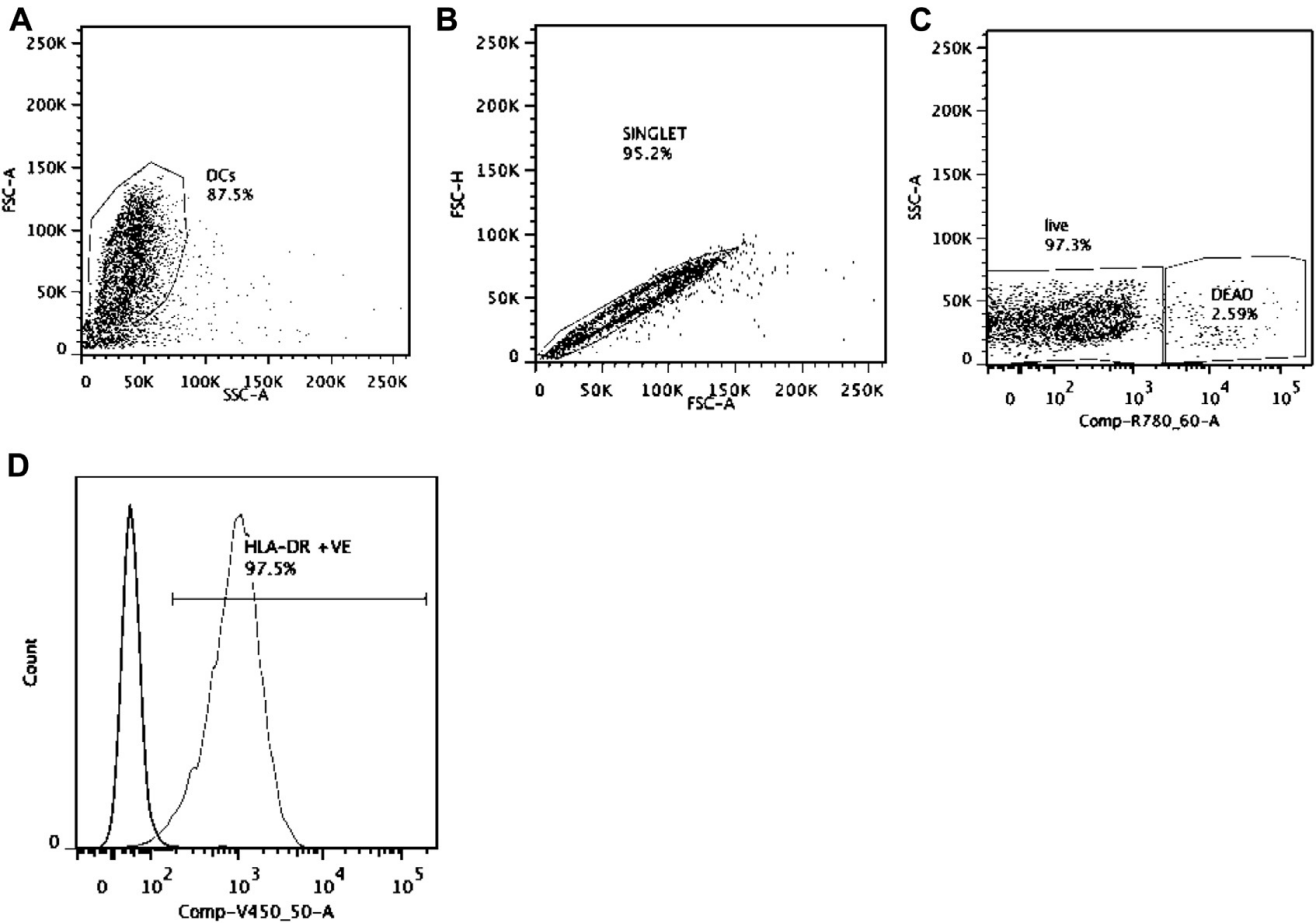
Gating strategy for cultured cells. **A,** Representative FlowJo cytometry plots show that forward scatter *(FSC)* versus side scatter *(SSC)* was used to draw a gate around the cells. **B,** Single cells were gated by plotting FSC height against FSC area to exclude doublets. **C,** Viable cells were gated by excluding dead cells stained with Ef780 dye. **D,** Single viable cells were then analyzed for individual markers, including HLA-DR *(lighter line)*, overlaid with their isotype controls. See Table E1 for full phenotypic details.

**Fig 4:**
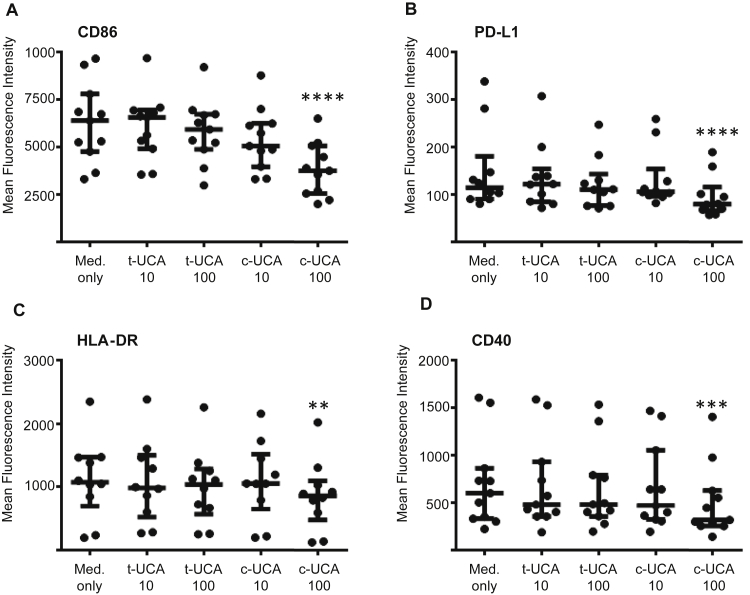
*Cis*-UCA reduces costimulatory molecule expression in unstimulated MDCCs. MDCCs were cultured for 7 days in the presence of *cis*-UCA *(c-UCA)* or *trans*-UCA *(t-UCA)*. Culture with 100 μg/mL *cis*-UCA caused a significant reduction in CD86 **(A)**, PD-L1 **(B)**, HLA-DR **(C)**, and CD40 **(D)** MFI. Statistical analysis was done with repeated-measures ANOVA (Friedman test with Dunn multiple comparison posttest). **P < .01, ***P < .001, and ****P < .0001. *Lines* show medians and interquartile ranges.

Although immature MDCCs at day 7 had only a low level of expression of HLA-DR and costimulatory molecules (see Table E1 and Fig E4 in this article's Online Repository at www.jacionline.org), cells conditioned with 100 μg/mL *cis*-UCA showed reduced expression of CD86, programmed death ligand 1 (PD-L1), HLA-DR, and CD40 compared with that seen in control cells (CD86 mean fluorescence intensity [MFI]: 3813 ± 1414 vs 6275 ± 2086, *P* < .0001; PD-L1 MFI: 96 ± 44 vs 149 ± 88, *P* < .0001; HLA-DR MFI: 863 ± 549 vs 1112 ± 627, *P* = .001; CD40 MFI: 507 ± 378 vs 708 ± 477, *P* = .0001; Fig 4), whereas conditioning with *trans*-UCA had no effect on expression of these molecules. Expression of CD1a and CD11c did not change with *cis*-UCA conditioning (data not shown).

**Fig 5:**
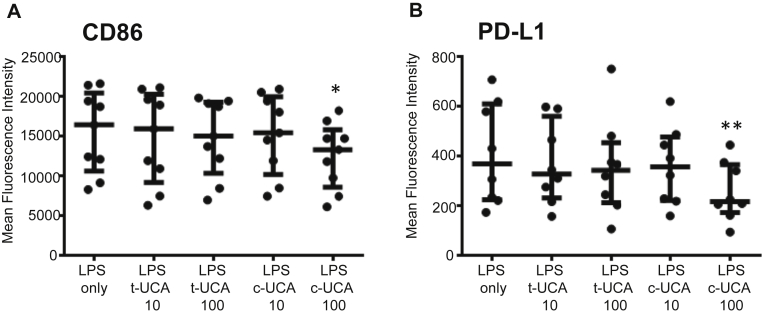
*Cis*-UCA reduces costimulatory molecule expression in LPS-stimulated MDCCs. MDCCs cultured for 7 days in the presence of *cis*-UCA *(c-UCA)* or *trans*-UCA *(t-UCA)* were stimulated with 1 ng/mL LPS for 24 hours. Conditioning with 100 μg/mL *cis*-UCA caused a significant reduction in CD86 **(A)** and PD-L1 **(B)** MFI. Statistical analysis was done with repeated-measures ANOVA (Friedman test with Dunn multiple comparison posttest). *P < .05 and **P < .01. *Lines* show medians and interquartile ranges.

MDCC stimulation with LPS or LTA on day 7 increased HLA-DR and costimulatory molecule expression on day 8, as expected (see Table E1). LPS-stimulated MDCCs conditioned with *cis*-UCA, but not *trans*-UCA, had significantly reduced expression of CD86 and PD-L1 compared with control values on day 8 (CD86 MFI: 12,541 ± 4,136 vs 15,490 ± 5,150, *P* = .04; PD-L1 MFI: 256 ± 118 vs 408 ± 204, *P* = .02; Fig 5). There were no significant changes in expression of HLA-DR, CD40, CD1a, or CD11c (data not shown). MDCCs stimulated with LTA showed a similar pattern of responses as LPS-stimulated cells (data not shown). Cells harvested at day 9 showed similar results to those harvested on day 7 or 8, indicating that *cis*-UCA did not simply delay maturation (data not shown).

### *Cis*-UCA–conditioned MDDCs induce regulatory T cells in coculture

**Fig 6:**
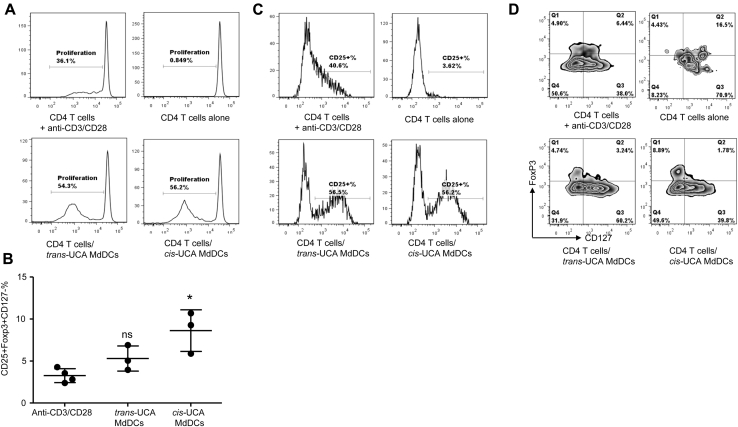
*Cis*-UCA–conditioned MDCCs induce more cells with a regulatory T-cell phenotype. **A,** Proliferation of CD4 T cells in coculture. **B,** Induction of cells with a regulatory T-cell phenotype after coculture. *ns*, Not significant. *P < .05, Kruskal-Wallis test with the Dunn multiple comparison posttest. **C,** Similar proportion of CD25^+^ cells after coculture with *trans*-UCA– and *cis*-UCA–conditioned MDCCs. **D,** More CD25^+^FoxP3^+^CD127^−^ cells after coculture with *cis*-UCA–conditioned MDCCs. In Fig 6, *A*, *C*, and *D*, Representative plots from 1 donor are shown.

*Cis*-UCA–conditioned immature MDCCs did not affect proliferation of CD4 T cells at day 5 compared with control values (Fig 6, *A*). However, CD4 T cells cocultured with *cis*-UCA–conditioned MDCCs showed a higher proportion of CD25^+^FoxP3^+^CD127^−^ cells at day 5 than those cultured with *trans*-UCA–conditioned MDCCs (Fig 6, *B-D*). This effect was not observed in CD4 T cells cocultured with *cis*-UCA–conditioned LPS-stimulated MDCCs (data not shown).

## Discussion

Although phenotypically different subsets of APCs have been identified in lesional AD skin, LCs are thought to be the only APCs found in nonlesional epidermis.^20^, ^21^ For many years, the classic paradigm of LC function was of antigen uptake and processing in the epidermis, followed by maturation and migration to skin draining lymph nodes for antigen presentation to T cells.^22^ This concept has recently been challenged by observations that the majority of cutaneous lymphocyte antigen–positive T cells are present in skin and that LCs can evoke both tolerogenic and immunogenic T-cell responses.^23^

The “outside-inside” theory of AD pathogenesis proposes that epidermal APCs in patients with AD will be exposed to more danger signals through a deficient skin barrier, leading to APC maturation and T cell–driven inflammatory skin disease. Using confocal microscopy, we confirmed that all CD1a^+^HLA-DR^+^ epidermal cells from suction blister roof samples express langerin, verifying that they are LCs. Although they are present in similar numbers, are of similar size, and have a similar number of dendrites per cell in each subject group, we demonstrate that LCs from *FLG*-null subjects both with and without AD have higher CD11c and CD83 expression than those of control subjects.

CD11c is a member of the CD18 integrin family and is expressed at a high level on most dendritic cells^24^ but only at a low level by LCs.^25^ It has previously been noted that some subjects have LCs expressing higher levels of CD11c in combination with increased HLA-DR expression.^26^ CD11c expression has been shown to be upregulated in human LCs treated with retinoic acid, and in the same experiment it was shown that a blocking antibody to CD11c was able to completely abrogate the ability of the LCs to present alloantigen to T cells, suggesting a critical role in antigen presentation.^27^ A recent study using confocal microscopy to observe the interaction between LCs and tight junctions in patients with AD also found occasional LCs with higher expression of HLA-DR, which they considered to be activated LCs.^28^ CD83 is a well-characterized marker of dendritic cell activation, which is expressed on mature LCs and might be involved in T-cell activation.^29^, ^30^ The upregulation of these molecules suggests that in nonlesional skin *FLG*-null subjects have more activated LCs compared with WT subjects.

In response to these findings, we investigated whether *FLG*-null subjects had a measurably deficient skin barrier, which could explain LC activation by increased exposure to danger signals. We found that *FLG*-null subjects with AD had a higher resting TEWL at baseline and required significantly fewer tape strips to increase TEWL to greater than 20 g/m^2^/h compared with WT subjects with AD (and control subjects), indicating an intrinsic upper epidermal fragility. These findings are in agreement with a recent study that used a similar tape-stripping method.^31^ Interestingly, however, not only did *FLG*-null subjects with normal skin show no difference in resting TEWL at baseline compared with control subjects, but also on physically challenging the epidermal barrier by means of tape stripping, they were again no different to control subjects. This implies that more than a genetic deficiency in filaggrin is required to produce a functionally deficient skin barrier.

To investigate why *FLG*-null subjects with physically normal-appearing skin could have more mature epidermal LCs, we studied the effects of the filaggrin breakdown product *cis*-UCA on MDCCs. Concentrations of UCA in human skin, as measured based on epidermal tape stripping, are known to vary widely between subjects, ranging from 2 to 62 nmol/cm^−2^ (equivalent to 40-1230 μg mL^−1^).^19^ A maximum of 60% to 70% of the total UCA concentration can be in the *cis* isomer at any time depending on recent UVR exposure,^32^ and both *cis*- and *trans*-UCA are able to diffuse into the subepidermal compartment (for more details, see the Methods section in this article's Online Repository).^32^, ^33^, ^34^, ^35^

We demonstrate that *cis*-UCA, but not *trans*-UCA, is able to downregulate the expression of CD86, HLA-DR, CD40, and PD-L1 on immature MDCCs and the expression of CD86 and PD-L1 on LPS-matured MDCCs. Our finding that immature MDCCs conditioned with *cis*-UCA induce a higher proportion of CD4 T cells with a regulatory T-cell phenotype in coculture is in agreement with the results of a previously published study.^36^ If *cis*-UCA is able to exert a similar effect on LCs, which are regarded as immature dendritic cells, *in vivo* this suggests a potential role in maintaining tolerance in the epidermis by preventing LCs from providing immunogenic signals to skin-resident T cells and by increasing their ability to induce a local regulatory T-cell phenotype. Additionally, because *cis*-UCA levels are increased by UVR, this is also potentially a mechanism for UVR-induced immunosuppression. Therefore a reduced level of *cis*-UCA, as would be expected in subjects with *FLG*-null mutations, could result in the LC maturation we observed in the epidermal suction blister samples.

**Fig E5:**
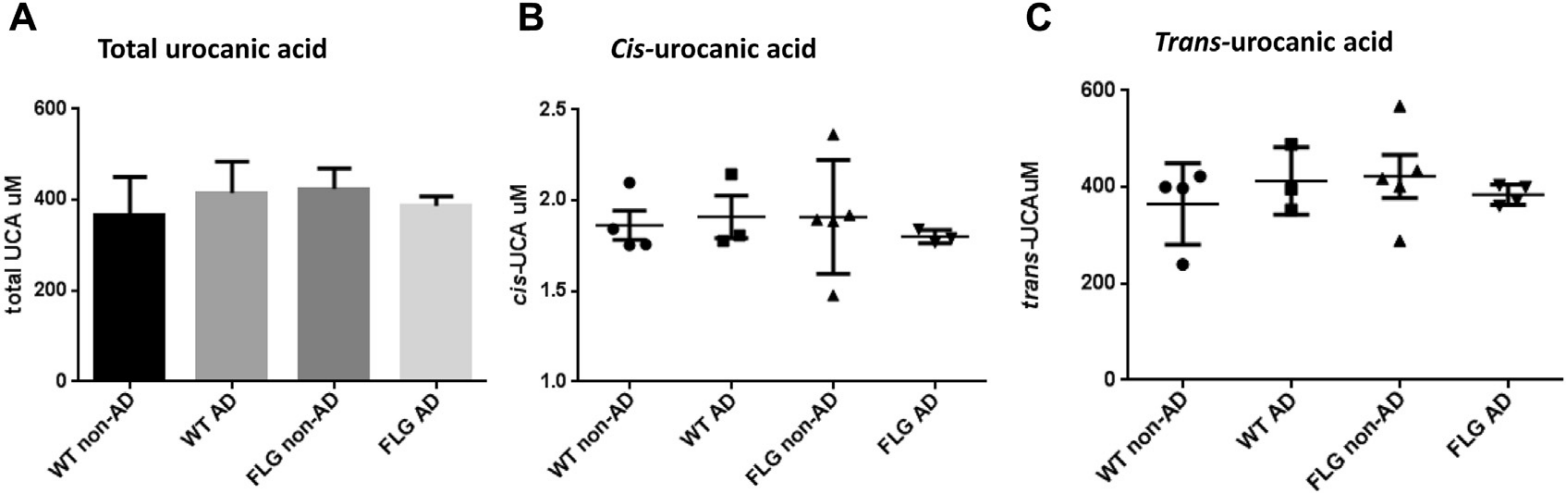
Concentration of UCA isomers in suction blister fluid. **A,** Bar chart comparing mean levels of UCA (*cis*-UCA and *trans*-UCA isomers combined) in suction blister fluid between different subject groups. The *narrow bar* shows SEMs. *P* = .66, Kruskal-Wallis test. **B,** Dot plot comparing mean levels of *cis*-UCA between different subject groups. *Bars* show means ± SEMs. *P* = .65, Kruskal-Wallis test. **C,** Dot plot comparing mean levels of *trans*-UCA between different subject groups. *Bars* show means ± SEMs. *P* = .66, Kruskal-Wallis test. Note the approximately 200-fold difference between *cis*- and *trans*-UCA concentrations.

Although we did not identify significant differences in levels of *cis*-UCA in suction blister fluid between the groups (see Fig E5 in this article's Online Repository at www.jacionline.org), a statistically significant stepwise decrease in total epidermal UCA levels from healthy control subjects to WT subjects with AD, *FLG*-null heterozygotes with AD, and *FLG*-null homozygotes with AD has been reported based on a tape-stripping method to determine UCA levels in the epidermis.^37^ Among patients with AD, *FLG* genotype was the major determinant of UCA levels, with disease severity as a secondary modifier.^37^

Our data do not address why some subjects with *FLG*-null mutations have AD and others maintain a normal clinical phenotype. If both groups of subjects have activated LCs in their epidermis, potentially providing activating signals to skin-resident T cells, it would suggest that there are overriding tolerogenic mechanisms in those without skin inflammation.

**Key messages**

• *FLG*-null subjects both with and without AD have more mature LCs in their epidermis than control subjects.
• The *cis*-isomer of UCA, a filaggrin breakdown product, is able to downregulate costimulatory molecule expression of dendritic cells and increase their induction of a regulatory T-cell phenotype in cocultures *in vitro.*
• Relative UCA deficiency in *FLG*-null subjects might be a mechanism for increased LC maturation and reduced epidermal regulatory T-cell populations, resulting in inflammatory skin disease.

## Figures and Tables

**Table E1:** Flow cytometric measurement of dendritic cell markers after 7 days of culture and after a further 24 hours of incubation in LPS

Marker	Day 7 (%)	Day 8, LPS 0.1 (%)	Day 8, LPS 1.0 (%)	MFI day 7	MFI day 8, LPS 0.1	MFI day 8, LPS 1.0
CD86	96 ± 4	99 ± 2	98 ± 2	6,275 ± 2,086	15,714 ± 4,383†	15,728 ± 4,637‡
CD40	91 ± 8	92 ± 1	96 ± 3	708 ± 477	564 ± 141	1,289 ± 6,964*
HLA-DR	96 ± 6	99 ± 1	98 ± 3	1,198 ± 661	1,289 ± 690	1,754 ± 877
PD-L1	34 ± 18	69 ± 26*	77 ± 28†	153 ± 85	401 ± 328	549 ± 377‡
CD80	61 ± 32	83 ± 14	83 ± 12	412 ± 206	617 ± 199	745 ± 231†
CD1a	62 ± 14	37 ± 20*	34 ± 22†	742 ± 363	273 ± 109†	409 ± 378*
CD14	2 ± 3	2 ± 2	2 ± 2	16 ± 22	9 ± 36	3 ± 43
CD11c	96 ± 4	99 ± 1	97 ± 4	5,517 ± 1,280	6,530 ± 1,890	7,384 ± 2,987

Data are presented as means ± SDs. CD86, CD40, HLA-DR, and CD11c were expressed by greater than 90% of cells at day 7. CD86 and CD40 increased in MFI after 24 hours of LPS stimulation. CD80 percentage showed a nonsignificant increase after LPS and a significant change in MFI after incubation in the higher LPS concentration. The immature dendritic cell marker CD1a was expressed by 61% of cells at day 7, but both percentage of positive cells and MFI decreased after incubation in LPS. The monocyte marker CD14 (expressed by all cells at the start of the culture period) was expressed at background levels at day 7 and was not altered by LPS stimulation. See the Methods section for culture details.

One-way ANOVA (Kruskal-Wallis test with the Dunn posttest) showed significant *P* values, as indicated: *P < .05, †*P* < .01, and ‡*P* < .001 compared with the value on day 7.

**Table I:** Clinical assessment of genotyped subjects

	WT non-AD	WT AD	*FLG* non-AD	*FLG* AD
TEWL and tape-stripping group				
No.	18	46	8	12
Age (y [range])	28 (19-59)	35 (18-76)	28 (19-58)	35 (22-57)
Male/female sex	8/10	16/30	4/4	5/7
SASSAD ± SD	NA	11 ± 9.3	NA	12 ± 7.5
IgE ± SD (IU/mL)∗	63 ± 63 (n = 14)	678 ± 1374 (n = 46)	33 ± 47 (n = 6)	443 ± 892 (n = 12)
Asthma	2 (11%)	15 (33%)	2 (25%)	5 (42%)
Hay fever†	1 (6%)	27 (59%)	1 (13%)	4 (33%)
Suction blister flow cytometry group				
No.	8	7	4	8
Age (y [range])	31 (20-55)	42 (27-76)	21 (19-25)	37 (27-57)
Male/female sex	3/5	3/4	3/1	3/5
SASSAD ± SD	NA	6 ± 4	NA	8 ± 3
Suction blister immunostaining group				
No.	4	3	5	4
Age (y [range])	34 (21-51)	21 (20-21)	35 (20-54)	40 (22-56)
Male/female sex	3/1	2/1	4/1	2/2
SASSAD ± SD	NA	12 ± 8	NA	15 ± 8

*NA*, Not applicable; *SASSAD*, Six-Area, Six-Sign Atopic Dermatitis score.

∗ *P* = .0001, AD versus non-AD.

† *P* = .0001, AD versus non-AD.

## Methods

### Antibody staining of epidermal APCs

Samples were stained with antibodies to CD1a (clone HI149–Alexa Fluor 700), CD11c (clone 3.9–Alexa Fluor 488), and HLA-DR (clone L243–phycoerythrin [PE]/Cy5) or isotype controls at 4°C for 30 minutes. Samples were then incubated with 1% SYTOX Blue Dead Cell Stain (Invitrogen). All antibodies were purchased from BioLegend (London, United Kingdom).

### Antibody staining panels for confocal microscopy of suction blister roofs

Antibody panel 1 was as follows: antibodies to CD1a (clone HI149–Alexa Fluor 647), CD1c (clone L161–Alexa Fluor 488), and CD207/langerin (clone 10E2) labeled with the Alexa Fluor 555 Monoclonal Antibody Labelling kit (Invitrogen A-20187) and CD11c (Clone 3.9) labeled with the Alexa Fluor 594 Monoclonal Antibody Labelling kit (Invitrogen A-20185). Antibody panel 2 was as follows: antibodies to HLA-DR (Clone L243–Alexa Fluor 647), CD83 (Clone HB15E–Alexa Fluor 488), and CD207/langerin (Clone 10E2) labeled with the Alexa Fluor 555 Monoclonal Antibody Labelling kit (Invitrogen A-20187), as before. Isotype control antibodies were used at identical concentrations. All samples were stained with 4′-6-diamidino-2-phenylindole dihydrochloride (DAPI; Invitrogen) 1:1000 for 3 minutes and then washed 3 times in PBS. All antibodies were purchased from BioLegend, unless otherwise stated.

### Antibody staining panels for flow cytometric analysis of MDDCs

Antibody panel 1 was as follows: lineage cocktail-1 (antibodies to CD3, CD14, CD16, CD19, CD20, and CD56–fluorescein isothiocyanate [FITC]) and antibodies to CD1a (clone HI149–Alexa Fluor 700, BioLegend), CD14 (clone MФP9–peridinin-chlorophyll-protein complex [PerCP]), CD40 (clone 5C3–APC, eBioscience), and CD86 (clone 2331-PE). Antibody panel 2 was as follows: antibodies to CD80 (clone L307.4–Alexa Fluor 700), CD11c (clone S-HCL-3–allophycocyanin), CD274/PDL (clone M1H1-1–PE), and HLA-DR (clone L243-ef450, eBioscience). The isotype antibodies IgG_1_ FITC (clone MOPC-21), IgG_2b_ FITC (clone MPC-11, BioLegend), IgG_1_ Alexa Fluor 700 (clone MOPC-21), IgG_2b_ PerCP (clone MPC-11, BioLegend), IgG_1_ APC (clone MOPC-21), IgG_1_ PE (clone MOPC-21), IgG_1_ ef450 (clone P3.6.2.8.1, eBioscience), and IgG_2a_ ef450 (clone eBM2a, eBioscience) were used at appropriate concentrations. EF780 viability dye (eBioscience) was used to exclude dead cells. All antibodies were purchased from BD Biosciences, unless otherwise stated.

### Isolation of CD4^+^ T cells from healthy volunteers

CD4^+^ T cells were isolated by means of negative selection with the human CD4^+^ T cell enrichment kit from STEMCELL (Manchester, United Kingdom), according to the manufacturer's instructions. CD14^−^ cells were resuspended at 5 × 10^7^ cells/mL in MACS buffer and incubated with 50 μL/mL EasySep Human CD4^+^ T Cell Enrichment Cocktail for 10 minutes at room temperature. Cells were then incubated with 100 μL/mL EasySep D Magnetic Particles for 5 minutes before being diluted with MACS buffer and incubated in Purple EasySep Magnet for 5 minutes. The untouched CD4^+^ T cells were poured off by inverting the magnet. A sample of cells was stained with an FITC-conjugated CD4 antibody to assess cell purity by using FACS analysis. Purity of the CD4^+^ T cells was consistently greater than 95%.

### Antibody staining of CD4^+^ T cells from mixed leukocyte reactions

Cells were stained with CD4 (clone RPA-T4–PerCP), CD25 (clone BC96–Alexa Fluor 488), CD127 (clone eBioRDR5–PE, eBioscience), and FoxP3 (clone 206D–Alexa Fluor 647). All antibodies were purchased from BioLegend, unless otherwise stated.

### Supplemental material: Concentration of UCA isomers in suction blister fluid

Both *cis*- and *trans*-UCA are able to diffuse into the subepidermal compartment. After UV exposure, *cis*-UCA is measurable in suction blister fluid for several weeks.E1, E2 It is also measurable in serum for 1 to 2 days^E3^ and urine for 2 weeks.^E4^ One study compared levels of epidermal UCA measured in blister roofs with levels of UCA in suction blister fluid in nonirradiated and irradiated human skin. In nonirradiated skin *cis*-UCA comprised 5% to 7% of epidermal UCA but was undetectable in blister fluid. In irradiated skin, however, it comprised 55% of epidermal UCA and 53% to 59% of blister fluid UCA.^E2^

Suction blister fluid samples were collected from the cohort of subjects who had suction blister roofs analyzed by using confocal microscopy (n = 16). Blister fluid was aspirated with a sterile needle attached to a 1-mL syringe, transferred to an Eppendorf tube, and stored at −80°C until samples were processed as a batch. Samples were analyzed by using high-performance liquid chromatography–mass spectrometry.

There was no significant difference in mean levels of UCA in suction blister fluid between groups, with mean concentrations ranging from 366 to 424 μmol/L (*cis*-UCA and *trans*-UCA isomers combined). The concentration of *cis*-UCA was approximately 200-fold less than the concentration of *trans*-UCA in all samples, making up only about 0.5% of the overall concentration of UCA in the suction blister fluid (Fig E5).

We hoped to demonstrate a correlation between *FLG* genotype and UCA levels in suction blister fluid from our subjects. Reported levels of UCA present in suction blister fluid from nonirradiated human skin range from 35 to 100 μmol/L, with corresponding levels of the *cis* isomer comprising 0% to 4% of the total UCA concentration.E1, E5 Levels of UCA in our suction blister fluid samples were around 400 μmol/L, which was 4 times higher than previously reported, whereas levels of *cis*-UCA comprised only about 0.5% of the total UCA present. Our samples were analyzed by using a different method (high-performance liquid chromatography–mass spectrometry compared with high-performance liquid chromatography alone), which might account for the difference in our results, and were also collected over the winter months in Scotland, which could result in a lower proportion of *cis*-UCA compared with *trans*-UCA. Because the concentration of UCA in our suction blister fluid samples was 10-fold less than that reported in epidermal tape-stripping samples, it might be that in nonirradiated skin any quantitative difference caused by a genetic mutation or inflammatory skin disease detectable at the skin surface is imperceptible by the time the small proportion diffuses into the dermal extracellular fluid sampled with the suction blister technique. Epidermal tape stripping would have been a better way to try and quantify differences in *cis*-UCA levels between our subject groups.
